# Regulation Systems of Bacteria such as *Escherichia coli* in Response to Nutrient Limitation and Environmental Stresses

**DOI:** 10.3390/metabo4010001

**Published:** 2013-12-30

**Authors:** Kazuyuki Shimizu

**Affiliations:** 1 Kyushu Institute of Technology, Fukuoka, Iizuka 820-8502, Japan; E-Mail: shimi@bio.kyutech.ac.jp; Tel.: +81-090-9729-9673; Fax: +81-948-29-7801; 2 Institute of Advanced Bioscience, Keio University, Yamagata, Tsuruoka 997-0017, Japan

**Keywords:** metabolic regulation, nutrient limitation, environmental stress, intracellular metabolite, global regulators, enzyme level regulation, transcriptional regulation

## Abstract

An overview was made to understand the regulation system of a bacterial cell such as *Escherichia coli* in response to nutrient limitation such as carbon, nitrogen, phosphate, sulfur, ion sources, and environmental stresses such as oxidative stress, acid shock, heat shock, and solvent stresses. It is quite important to understand how the cell detects environmental signals, integrate such information, and how the cell system is regulated. As for catabolite regulation, F1,6B P (FDP), PEP, and PYR play important roles in enzyme level regulation together with transcriptional regulation by such transcription factors as Cra, Fis, CsrA, and cAMP-Crp. αKG plays an important role in the coordinated control between carbon (C)- and nitrogen (N)-limitations, where αKG inhibits enzyme I (EI) of phosphotransferase system (PTS), thus regulating the glucose uptake rate in accordance with N level. As such, multiple regulation systems are co-ordinated for the cell synthesis and energy generation against nutrient limitations and environmental stresses. As for oxidative stress, the TCA cycle both generates and scavenges the reactive oxygen species (ROSs), where NADPH produced at ICDH and the oxidative pentose phosphate pathways play an important role in coping with oxidative stress. Solvent resistant mechanism was also considered for the stresses caused by biofuels and biochemicals production in the cell.

## 1. Introduction

Living organism must survive in response to a variety of environmental perturbations. For this, living organisms sense environmental changes by detecting extracellular signals such as the concentrations of nutrients such as carbon, nitrogen, phosphate, sulfur, ion sources, and the growth conditions such as pH, temperature, oxygen availability or oxidative stress, osmotic stress, and solvent stress. These signals eventually feed into the transcriptional regulatory systems, which affect the physiological and morphological changes that enable organisms to adapt effectively for survival [1].

Since environmental perturbations occur simultaneously, cells must recognize the changes, integrate such information, and adjust the metabolism systematically. To understand how the cell system regulates for the variety of perturbations, or how the cells integrate such information, local molecular knowledge alone may not be sufficient. Instead, it is important to understand how the cell system’s behavior emerges from interactions between characterized molecules [2,3]. To understand such mechanisms, the coupling between recognition and adjustment aspects, and between enzyme regulation and gene level regulation must be understood in the network context [4].

Biological systems are known to be robust and adaptable to culture environment. Such robustness is inherent in the biochemical and genetic networks. Several genes that are necessary to respond to various environmental or nutritional changes require specific recognition by RNA polymerase associated with alternative sigma factors. Here, we consider how environmental changes are detected through signaling systems by modulating appropriate transcription factors, how the metabolic pathway genes are regulated by the corresponding transcription factors, and how the metabolism changes.

Living organisms such as bacterial cells have complex but efficient mechanisms to respond to the change in culture environment. This is mainly achieved by the so-called global regulators, where they generally act at transcriptional level. A two-component signal transduction system is considered to be the important means of detecting extracellular signals and transducing the signals into cytosol for metabolic regulation. These involve a phospho-relay from a transmembrane histidine protein kinase sensor to the target response regulator. In the case of *E. coli*, 29 transcription factors (TFs) show such regulation with 28 histidine protein kinase [5], where the genes encoding the two components are usually located within the same operon, enabling their coordinated expression. Note that there might exist a cross-talk between noncognate sensors and regulators [6].

**Figure 1:**
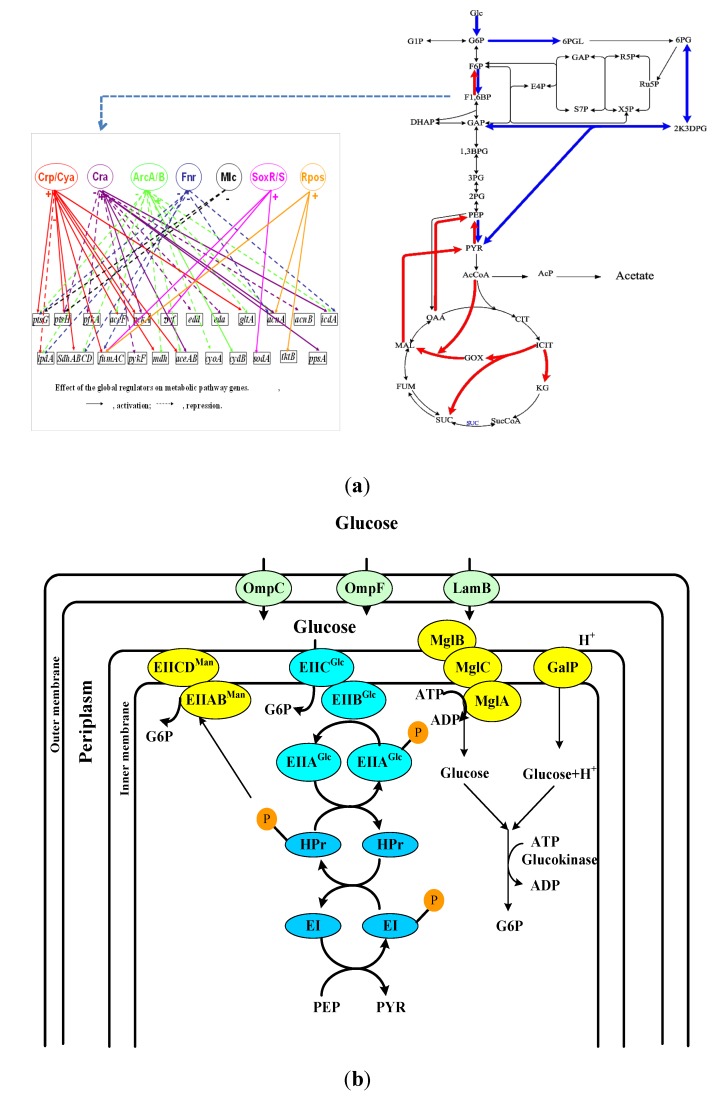
Main metabolic pathways and its regulation: (**a**) Control by TFs such as Cra; and (**b**) transport of glucose *via* PTS and non-PTS transporters.

In addition to exogenous signals, the cell can recognize the cell’s state by detecting the intracellular metabolites. The typical example is catabolite repressor/activator protein Cra (originally called fructose repressor protein FruR) which binds a key intermediate such as fructose 1,6-bis phosphate (F1, 6BP or FDP) and regulates the carbon flow (Figure 1a). FDP plays an important role in flux sensing, which will be mentioned later. There exists a hybrid type of TFs where they sense the metabolites that are transported from the culture environment or synthesized endogenously. This can typically be seen in regulating amino acid synthetic pathways, possibly because it is preferable for the cell to import essential metabolites when they are freely available rather than expend energy on their production [1].

In relation to global regulators, sigma factors also play important roles, where they allow RNA polymerase to be recruited at specific DNA sequences in the promoter regions at which they initiate transcription. In *E. coli*, seven sigma factors have been found so far, and these play important roles depending on the environmental stimuli (σ^19^: ion transport, σ^24^: extreme temperature, σ^28^: flagella genes, σ^32^: heat shock, σ^38^: stationary phase or carbon starvation *etc.*, σ^54^: nitrogen regulation, σ^70^: house keeping) [7]. H-NS (histone-like nucleotide structuring protein) is another type of global transcriptional regulator, which regulates a variety of physiological functions such as metabolism, fimbriae expression, virulence flagella synthesis, and proper function [8].

Other types of global regulators are signaling molecules such as cyclic-AMP (cAMP) and cyclic-di-GMP (bis-(3'-5')-cyclic-dimeric guanosine monophosphate) [9,10]. The cAMP is synthesized from ATP by Cya (adenylate cyclase) at low glucose concentration with an increase in phosphorylated EIIA^Glc^ (EIIA^Glc^-P) involved in phosphotransferase system (PTS)(Figure 1b), where EIIA^Glc^-P activates Cya activity. The cAMP binds to Crp (cAMP receptor protein), also known as CAP (catabolite activation protein), and cAMP-Crp complex becomes an activated transcription factor in relation to catabolite regulation as will be explained in more detail later. Note that cAMP regulates not only catabolite regulation, but also flagellum synthesis, biofilm formation, quorum sensing, and nitrogen regulation [10,11,12,13].

As another level of regulation, small noncoding RNAs (sRNAs) play important roles in the post-transcriptional regulation [14]. The sRNAs are mainly involved in stress response regulation, pathogenesis, and virulence. A single sRNA can affect multiple targets, where sRNAs modify the translation or stability of the targets and chaperone. One such example is SgrS in *E. coli,* where it binds to mRNA of *ptsG* gene, which encodes EIICB^Glc^ for glucose uptake [15]. Another group of sRNAs bind to proteins, where such example is CsrB in *E. coli* [16], and these sRNAs regulate the activity of CsrA, a global regulator for carbon storage regulation. The sRNAs are involved in the fine-tuning of gene expression by binding to target mRNAs with mediation of the RNA chaperon Hfq [17,18]. This expression depends on the specific environmental conditions such as oxidative stress, cell envelope homeostasis, and glucose starvation [19].

Metabolic regulation mechanism is quite complex [20,21], but a wide variety of data are accumulating together with molecular and biological knowledge, and it is desirable to appropriately understand the regulation mechanism of the whole cell system. Below, an attempt is made to overview the regulation mechanism in response to the variety of culture environmental perturbations, keeping in mind the basic schemes as mentioned above for bacterial cells, in particular for *E.coli.*

## 2. Transport of Nutrient Molecules and PTS

Microorganisms must uptake nutrients from the external environment, where it is critical to transport the nutrients rapidly across cell membranes and maintain enough nutrient levels in the cytoplasm. Some of the nutrients, such as CO_2_ or NH_3_, can diffuse across membrane somewhat faster than water [22,23]. The driving force for such transport is the concentration differences between cytosol and external environment. Large membrane permeability gives the benefit of high ambient nutrient levels, where passive diffusion alone can supply enough nutrients for the cell growth without spending energy. On the other hand, if ambient nutrient level is low, intracellular nutrient may diffuse out, and thus active transport system must function to pump in scarce nutrient present in the external environment for survival. Those examples are AmtB to capture scarce ammonium, Pst system to capture scarce phosphate, as will be explained later in more detail.

The gram-negative bacteria such as *E. coli* have outer membrane and inner cytoplasmic membrane, which act as hydrophobic barrier against polar compounds. The outer membrane contains channel proteins, where the specific molecules can only move across these channels. In the outer membrane of *E. coli*, 108 channels are formed by porin proteins [24], These are open but regulated water-filled pores that form substrate-specific, ion-selective, or nonspecific channels that allow the influx of small hydrophilic nutrient molecules and the efflux of waste products [25]. They also exclude many antibiotics and inhibitors that are large and lipophilic [26]. The OmpC and OmpF are the most abundant porins present under typical growth conditions [27]. Their relative abundance changes depending on osmolarity, temperature, and growth phase. Under glucose limitation, outer membrane glycoporin LamB is induced [28], where this protein permeates such carbohydrates as maltose, maltodextrins, and glucose [29].

**Figure 2:**
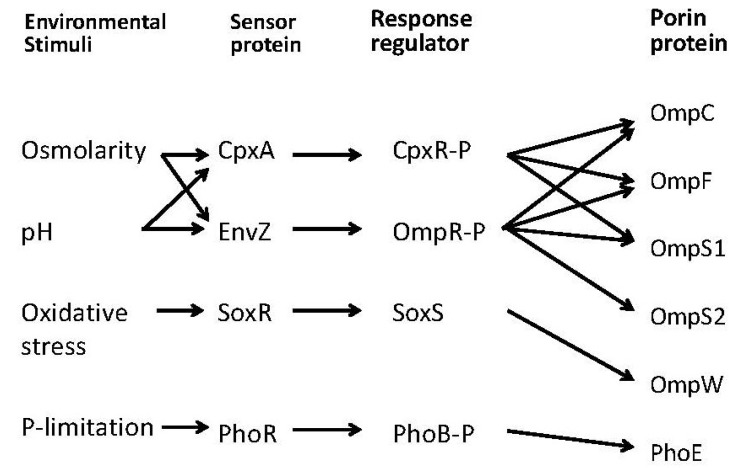
Regulation of porin genes in response to culture environment.

Porin genes are under the control of the two-component EnvZ-OmpR system, where EnvZ is an inner membrane sensor kinase and OmpR is the cytoplasmic response regulator. Figure 2 shows how porin genes are regulated by two-component systems. In response to osmolarity, pH, temperature, nutrients, and toxins, EnvZ phosphorylates OmpR, where the phosphorylated OmpR (OmpR-P) regulates porin genes [25]. Acetyl phosphate (AcP) can function as a phosphate donor for OmpR under certain condition. OmpR controls cellular processes such as chemotaxis and virulence as well [30]. In terms of virulence, abolition of porin formation diminishes pathogenesis [25].

The first step in the metabolism of carbohydrates is the transport of these molecules into cytosol. In bacteria, various carbohydrates are taken up by several mechanisms [31]. The most common mechanism is the transport by phosphotransferarse system (PTS), where glucose can be transported from periplasm into cytosol, where PTS is widespread in bacteria and absent in archaea and eukaryotic organisms [32]. PTS is composed of soluble and nonsugar-specific components, Enzyme I (EI) encoded by *ptsI* and phosphohistidine carrier protein (HPr) encoded by *ptsH*, where they transfer phosphoryl group from phosphoenol pyruvate (PEP) produced in the main metabolism to the sugar-specific enzyme IIA and IIB. Another component of PTS is enzyme IIC (in some cases also IID) which is an integral membrane protein permease that transports sugar molecules, where it is phosphorylated by EIIB (Figure 1b). So far, 21 different EII complexes have been identified in *E. coli*, and those are involved in the transport of about 20 different carbohydrates such as glucose, fructose, mannose, mannitol, galactitol, sorbitol *etc.* [33]. In *E. coli*, glucose can be transported by EII^Glc^ and EII^Man^. The EII^Glc^ is composed of soluble EIIA^Glc^ encoded by *crr* and of integral membrane permease EIICB^Glc^ encoded by *ptsG.*

In *ptsG* mutant, glucose can be transported by EII^Man^ complex, and the cell can grow with less growth rate than the wild-type strain [34]. Under glucose limitation, *galP* is induced, where it codes for low-affinity galactose: H^+^ symporter GalP. The genes in the *mglABC* operon encode an ATP-binding protein, a galactose/glucose periplasmic binding protein, and an integral membrane transporter protein, respectively, forming Mgl system for galactose/glucose import [35] (Figure 1b). When extracellular glucose concentration is very low, the Mgl system together with LamB attains high-affinity glucose transport [35]. The glucose molecule transported either by the GalP or Mgl systems must be phosphorylated by glucokinase (Glk) encoded by *glk* from ATP to become glucose 6-phosphate (G6P) [36] (Figure 1b).

The non-PTS carbohydrates such as xylose, glycerol, galactose, lactose, arabinose, rhamnose, maltose, melibiose, and fucose are recognized through TFs. Neither trans-membrane sensors nor regulatory proteins with sensing function have been identified so far for organic acids such as acetate, succinate, or malate, and it is unclear how these carbon sources are recognized [4], while formate is transported by Foc.

## 3. Flux Sensor

**Figure 3:**
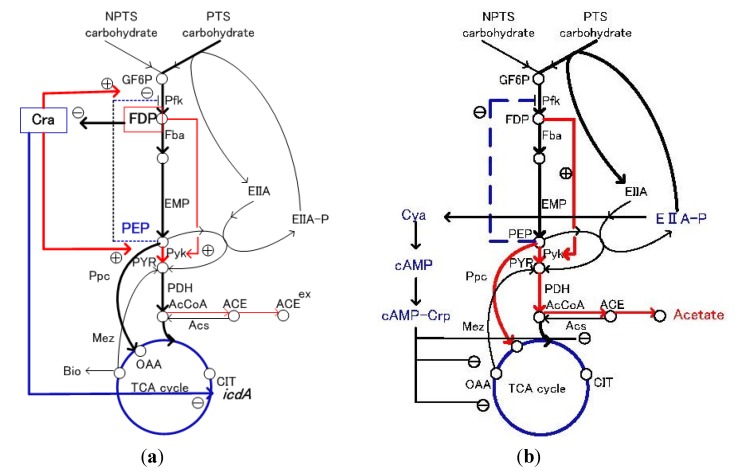
Feedforward and feedback controles by enzyme level and transcriptional regulations: (**a**) regulation by Cra, and (**b**) regulation by cAMP-Crp.

In addition to the canonical nutrient sensors, which measure the concentrations of nutrients, the concept of flux sensor may be useful as a novel impetus for metabolic regulation, where the metabolic fluxes may be sensed by molecular systems as flux sensors [4,37,38]. Namely, if there is a strong (linear) relationship between the specific flux and the specific metabolite concentration, flux changes can be detected by the corresponding metabolite concentration. For example, the fluxes of lower glycolysis and the feedforward activation of FDP on Pyk show such characteristics (Figure 3a). Moreover, the interaction of this flux-signaling metabolite with Cra then leads to flux-dependent regulation (Figure 3a). Instead of utilizing nutrient specific receptors to sense the environmental signals, which require the simultaneous expression of a large number of receptors, and impose a large burden on the cell, the flux-sensing system simply recognizes the fluxes by the intracellular metabolite as integral signal. Since the relationship between FDP and the lower part of glycolysis flux depends on the allosteric regulation of FDP on Pyk, this relationship may not hold for certain mutants such as *pyk* mutant.

**Figure 4:**
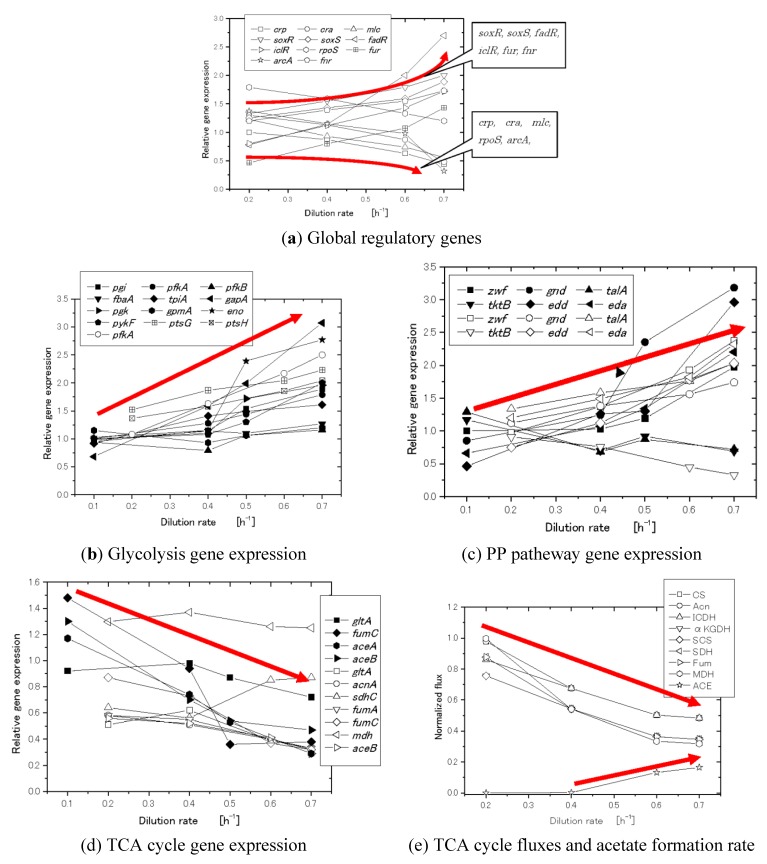
Effect of the growth rate (dilution rate in the continuous culture) on gene expression and fluxes: (**a**) global regulators, (**b**) glycolysis genes, (**c**) TCA cycle genes, (**d**) PP pathway genes, and (**e**) TCA cycle fluxes and acetate formation rate. Open symbol from [47], while filled symbol from [48].

In general, there might be a correlation between a certain metabolic flux and a particular phenotype based on flux-dependent regulation. For example, there is a relationship between the specific glucose uptake rate and the activities of the respiratory and fermentative pathways. Namely, there is a correlation between the specific glucose uptake rate and the ethanol production rate in the cultivation of *Saccharomyces cerevisiae*, whereas there is no correlation between the specific growth rate and the ethanol production rate [37]. In the case of *E.coli*, acetate production has been considered to be an overflow metabolism with respect to the specific growth rate (or the dilution rate in the continuous D-stat culture or accelerating A-stat culture) [39,40,41]. As far as wild type strain is considered, the specific growth rate is correlated with the specific glucose uptake rate by the yield coefficient [42]. However, if we consider the case of nitrogen limitation [43], the specific acetate production rate is not correlated with the cell growth rate, but may correlate with the specific glucose uptake rate. As the specific glucose uptake rate increases in accordance with the specific growth rate, the phosphorylated EⅡA^Glc^ (EⅡA^Glc^-P) decreases [44,45], which in turn decreases the activity of Cya, and cAMP level as well as cAMP-Crp level decreases (Figure 3b). The decreased cAMP-Crp represses the glucose uptake rate by the decreased expression of *ptsG*, which forms the feedback regulation to the initial increase in the glucose uptake rate [46] (Figure 3). Figure 4 shows how the categorized gene expression changes with respect to the dilution rate (the specific growth rate) [47,48]. Figure 4a abd 4c shows that the decrease in cAMP-Crp transcriptionally represses the TCA cycle gene expression as the cell growth rate or the specific glucose consumption rate increases [39,47,48]. Although the TCA cycle gene expression is repressed, and the relative flux in % decreases with respect to the specific glucose consumption rate [41,47] (Figure 4e), the absolute flux on the mmol basis may increase due to increased glucose up-take rate [47]. Consider why the TCA cycle has to be transcriptionally repressed. As mentioned above, the specific NADH production rate increases as TCA cycle activity increases, which in turn activates the respiration as far as enough oxygen is available. However, this causes a high possibility of producing oxygen radicals, which gives the critical damage to the cell, where this is protected by such transcription factors as OxyR and SoxR/S affecting the expression of such genes as *sodA* and *catA* as will be explained later in the section of oxidative stress. Thus, it may be presumed that the TCA cycle has an upper bound to avoid the production of oxygen radicals. Note that the minimum TCA cycle flux is always needed to supply αketo glutarate (αKG) for ammonium assimilation *via* glutamate dehydrogenase (GDH) to form glutamate (and also glutamine), and then the nitrogen source (N) is spread around other amino acids by subsequent transanimations.

It may be considered that the dominance of catabolite regulation overrides many other regulation processes *via crp*, where the most relevant control mechanism is cAMP-dependent catabolite repression of the PEP-glyoxylate cycle with low TCA cycle fluxes based on ^13^C-flux analysis of *E. coli* mutants [42]. In the *cya* and *crp* mutants that lack cAMP-Crp complex, glyoxylate shunt activity is essentially abolished, and such cycle will not appear.

In the case of carbon starvation, AMP and PEP increase for both *E.coli* and Yeast [49]. Note that cAMP is not the starvation signal for Yeast, while it is for *E.coli*. AMP may reflect energy limitation for both organisms, where AMP is in equilibrium with ADP to ATP ratio by adenylate kinase Adk [49]. In both organisms, PEP level increases under carbon starvation, where Pyk is allosterically deactivated by the decrease in FDP, a positive feedforward regulator as mentioned above (Figure 3a). The increased PEP in turn inhibits Pfk activity, reinforcing the blockage of glycolysis, and then reduces the glucose uptake rate. In the case of *E.coli*, the decrease in FDP further activates Cra activity, and transcriptionally represses *pfkA* and *pykF* gene expression (Figure 3a). Moreover, the increase in PEP concentration and thus PEP/PYR ratio increases phosphorylation of EⅡA and activates Cya, resulting in the increase in cAMP level in *E.coli*, while it is kept low in the case of Yeast.

One of the most important metabolic rearrangements is the transition from glycolytic to gluconeogenetic carbon sources that involve redirection of the carbon flow through the main metabolic pathways at the late growth phase or early stationary phase in the batch culture. This phenomenon is wide-spread to various organisms. This diauxic shift in *S. cerevisiae* is accomplished by the following three events: (1) a reduction in the glycolytic flux with production of storage compounds before glucose depletion, (2) upon glucose exhaustion, the reversion of carbon flow through glycosis with activation of the glyoxylate pathway, and (3) shutting down of the pentose phosphate (PP) pathway with a change in NADPH regeneration in the later stages [50].

## 4. Carbon Storage Regulation

The carbon storage regulator (Csr) system influences a variety of physiological processes such as central carbon metabolism, biofilm formation, motility, peptide uptake, virulence and pathogenesis, quorum sensing, and oxidative stress response [8,51,52,53]. Csr is controlled by the RNA-binding protein CsrA, a posttranscriptional global regulator that regulates mRNA stability and translation [54,55,56], where CsrA is regulated by two sRNAs such as CsrB and CsrC [57,58,59]. CsrA represses glycogen accumulation by regulating the expression of *glgCAP* operon and *glgB* of *glgBX* operon [8,60]. CsrA regulates the central carbon metabolism and glycogenesis such that glycogen synthesis pathway genes, as well as gluconeogenic pathway genes are repressed, while glycolysis genes are activated [8,61] (Supplementary Table S1).

From the practical application point of view, phenylalanine production can be enhanced by manipulation of Csr, where *csrA* mutation causes a significant increase in intracellular PEP concentration, since CsrA positively regulates *pykF*, while negatively regulates *pckA* and *ppsA*. The precursor of shikimate pathway for aromatic amino acids production are a single E4P and two PEP molecules, and thus over-expression of *tktA* with *csrA* gene disruption enhances phenylalanine biosynthesis [62].

The biofuels production can be improved by the over-expression of CsrB by activating native fatty acid and heterologous n-butanol and isoprenoid pathways [61]. In particular, CsrB-mediated degradation of CsrA drives over-expression of *glgCAP* operon, which results in the accretion of the storage polysaccharide glycogen. AcCoA and several amino acid concentrations increase with concurrent decrease in acetate level with this mutation [61]. Moreover, CsrB impacts the expression of the stringent response regulator DksA, where it is responsible for transcriptional activation of CsrB during stringent response [63]. This suggests that CsrB directly regulates DksA through CsrA, thereby forming a positive feedback loop, indicating the links between CsrA/B and the stringent response [61,63].

## 5. Nitrogen Regulation

**Figure 5:**
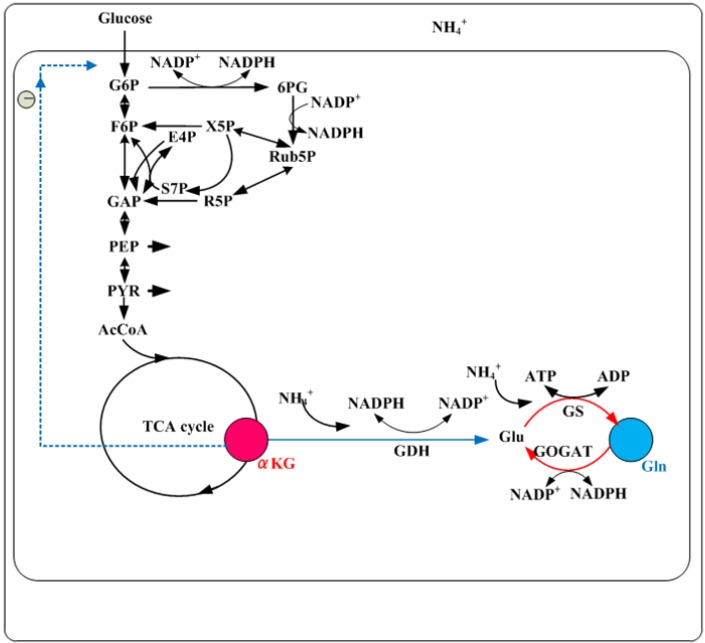
NH_3_ assimilation pathways and the regulation under N-limitation.

Next to carbon (C) source metabolism, nitrogen (N) metabolism is also important in understanding the metabolic regulation. In *E. coli*, assimilation of N-source such as ammonia/ ammonium (NH_4_^+^) using 𝛼-KG results in the synthesis of glutamate and glutamine (Figure 5). Glutamine synthetase (GS, encoded by *glnA*) catalyzes the only pathway for glutamine biosynthesis. Glutamate can be synthesized by two pathways through the combined actions of GS and glutamate synthase (GOGAT, encoded by *gltBD*) forming GS/GOGAT cycle, or by glutamate dehydrogenase (GDH encoded by *gdhA*) [64] (Figure 5). When extracellular NH_4_^+^ concentration is low around 5 μM or less, ammonium enters into the cell *via* AmtB, and is converted to glutamine by GS, and UTase (uridylyl transferase) uridylylates both GlnK and GlnB [65](Figure 5). When extracellular NH_4_^+^ concentration is more than 50 μM, the metabolic demand for glutamine pool rises, and UTase deuridylylates GlnK and GlnB. GlnK complexes with AmtB, thereby inhibiting the transport *via* AmtB, where GlnB interacts with NtrB and activates its phosphatase activity leading to dephosphorylation of NtrC, and NtrC-dependent gene expression ceases [65].

GlnK binds to AmtB and inhibits its activity under ammonium rich conditions, while GlnK dissociates from AmtB at low ammonium concentration, thereby setting AmtB free to act for ammonium transport [66,67,68] (Figure 5). Intracellular ammonium is assimilated into biomass in two steps: Namely, it is first captured in the form of glutamic acid (Glu) using carbon skeleton of αKG *via* GS/GOGAT cycle. Then N-group in Glu is transferred to synthesize amino acids thus incorporating into biomass, while recycling the carbon skeleton back to αKG [69]. The αKG pool, which integrates imbalance between the ammonium assimilation flux and the biomass incorporation flux activates AmtB [66,67,68] *via* GlnK. If the internal ammonium level drops, then the rate of ammonium assimilation will drop immediately. This results in αKG accumulation [70] (Figure 5).

From the studies on interdependence of different metabolic routes, two of the major signal transduction systems of N and C metabolisms have been identified as P_II_, a small nitrogen regulatory protein and PTS. Because of the important roles in the regulatory functions, P_II_ and PTS can be regarded as the central processing units of N and C metabolisms, respectively. The P_II_ protein senses 𝛼KG and ATP, thus link the state of central carbon and energy metabolism for the control of N assimilation [71]. The glucose catabolism is modulated by the global regulators such as Cra, Crp, Cya, and Mlc as mentioned before, while N assimilation is regulated by P_II_-Ntr system together with Crp, providing a regulatory network between C and N assimilation in *E. coli* [72]. The C and N metabolisms may be linked by energy metabolism, where P_II_ protein controls N assimilation by acting as a sensor of adenylate energy charge, which is the measure of energy available for the metabolism. Moreover, 𝛼KG serves as a cellular signal of C and N status, and strongly regulates P_II_ functions [73]. Gln and αKG are the signal metabolites for nitrogen and carbon status, respectively, and these signals regulate GS adenylylation state and nitrogen regulator I (NR_I_ or NtrC) phosphorylation state [74]. Nitrogen shortage is reflected by the reduced Gln levels and increased αKG level [49,70] (Figure 5). While the ratio of Gln to αKG varies in response to N-limitation, this ratio is substantially constant under C-limitation. This constant ratio requires tuning the rate of ammonia assimilation to match exactly the carbon uptake rate. This ratio is insensitive to variations in protein levels of the core circuit and to the N-utilization rate, and this robustness depends on bifunctional enzyme adenylyl transferase [75].

During N-limitation, a sudden increase in nitrogen availability results in immediate increase in glucose uptake, and αKG plays an important role for this, where αKG directly blocks the glucose uptake under N-limitation by inhibiting EI of PTS [76] (Figure 5). This implies several things: (1) αKG inhibition of sugar uptake is for all PTS sugars by inhibiting EI but not carbohydrate specific E, (2) this is performed without perturbing the concentrations of the glycolytic intermediates such as G6P, PEP, and PYR, (3) inhibition of EI by αKG leads to reduced amount of phosphorylated EⅡAGlc and decreases cAMP level, where the effect of αKG on cAMP production is caused by the difference in EⅡA^Glc^ phosphorylation rather than a difference in substrate availability [76]. Moreover, αKG is a promiscuous enzymatic regulator that competitively inhibits CS of the TCA cycle and 3PG dehydrogenase for serine biosynthesis, and further controls aspartate production by product inhibition of transaminase under N-limitation [76]. Note that αKG noncompetitively inhibits EI and Pps, while PtsP (EI homolog in the nitrogen PTS, which will be mentioned next) is insensitive to αKG.

## 6. The Role of PTS^Ntr^

In addition to carbohydrate PTS, most proteobacteria possess a paralogous system such as nitrogen phosphotransferase system PTS^ Ntr^, where it consists of EI^Ntr^ encoded by *ptsP*, NPr encoded by *ptsO*, and EⅡA^Ntr^ encoded by ptsN, which are paralogues of the carbohydrate PTS components such as EI, HPr, EⅡA, respectively [77,78,79].

E.coli PTS^Ntr^ plays a role in relation to K^+^ uptake, where dephosphorylated EⅡANtr binds to and regulates the low affinity K^+^ transporter TrkA [80] and the K^+^-dependent sensor kinase KdpD [79,81]. K^+^ regulates global gene expression involving both σ^70^- and σS-dependent promoters [82]. Moreover, dephosphorylated EⅡA^Ntr^ modulates the phosphate starvation response through interaction with sensor kinase PhoR [83]. The dephosphorylated form of NPr interacts with and inhibits LpxD, which catalyses biosynthesis of lipidA of the lipopolysaccharide (LPS) layer [84].

Although the physiological role of Ntr has not been well known, glutamine and αKG, reciprocally regulate the phosphorylation state of the PTS^Ntr^ by direct effects on EI^Ntr^ auto-phosphorylation. This implies that PTS^Ntr^ senses nitrogen availability [85].

## 7. Regulation for S-limitation

At least three metabolites such as (1) sulfide, the reduction product of sulfate, which is used for cystein biosynthesis, (2) N-acetylserine, the only precursor of cysteine, and (3) adenosine 5'-phosphosulphate (APS), the first intermediate in sulfate assimilation are involved in perceiving S-limitation [86,87]. The concentrations of sulfide and APS decrease, while N-acetylserine pool increases under S-limitation. The two regulators CysB and Cbl mediate homeostatic responses to S-limitation, where these responses help *E.coli* to scavenge trace amounts of cystein and sulfate, preferred S sources, or the alternative S sources such as glutathione and various alkaline sulfonate including taurin. S-limitation affects methionine metabolism, synthesis of FeS clusters, and oxidative stress.

Like NtrC for N-regulation, CysB is the primary regulator for homeostatic responses to S, and it is required for the synthesis of Cbl [88]. CysB is positively controlled by N-acetylserine and negatively controlled by sulfide or thiosulfate [87], and Cbl is negatively controlled by APS [86]. It is of interest that *cbl* gene is transcribed from *nac* promoter under N-limitation [89]. The *ddp* operon is activated by NtrC, and there might be a cross regulation between S-limitation and N-limitation [90].

## 8. Phosphate Regulation

The phosphate (P) metabolism is also quite important from the energy generation and phosphorelay regulation points of view. The phosphorous compounds serve as major building blocks of many biomolecules and have important roles in signal transduction [91]. Depending on the concentration of environmental phosphate, *E. coli* controls phosphate metabolism through Pho regulon, which forms a global regulatory circuit involved in a bacterial phosphate management [91,92]. The PhoR/PhoB two-component system plays an important role in detecting and responding to the changes of the environmental phosphate concentration [93].

When cells enter into P_i_-starvation phase in the batch culture, the Pho regulon is activated, and σ^S^ starts to accumulate in the cytosol [91,94,95]. The promoters of the Pho genes are recognized by σ^D^-associated RNA polymerase. A mutation in *rpoS*, significantly increases the level of AP (alkaline phosphatase) activity, and the overexpression of σ^S^ inhibits it [96]. Other Pho genes such as *phoE* and *ugpB* are likewise affected by σ^S^. The RpoS may inhibit the transcriptions of *phoA, phoB, phoE,* and *ugpB*, but not that of *pstS* [96]. The *pst* may be transcribed by both σ^S^ and σ^D^. The Pho regulon is thus evolved to maintain a tradeoff between cell nutrition and cell survival during P_i_-starvation [96].

The Pho regulon and the stress response may be interrelated [95,96,97,98,99,100]. The presence of glucose or mutations in *cya* or *crp* leads to the induction of *phoA* gene in *phoR* mutatnt. This induction requires the sensor PhoM (CreC) and the regulator PhoB [101].

One P_i_-independent control is the regulation by the synthesis of acetyl phosphate (AcP), where P_i_ is incorporated into ATP at Ack (acetate kinase) pathway. AcP may then act indirectly on PhoB.

## 9. Regulation for Ion Uptake

The metal levels are often sensed by metal-sensing regulatory RNA, which encodes metal-sensing proteins involved in the transport and storage of intra-cellular metals [102,103]. In the native environment, the cell such as *E. coli* continuously faces iron deficiency, where metal ion functions as cofactor in many of the cellular constituents such as flavoproteins, and therefore, the cell furnishes the mechanism for iron uptake and storage system [104,105]. However, excess iron causes toxicity by catalyzing the formation of reactive free radicals through Fenton/ Haber-Weiss reaction [106]. Aerobic respiration generates superoxide ions (O_2_^−^), with NDH II as the main generator of endogenous superoxide and NDH I and SDH as small contributors [107]. In combination with inability to convert NADH to NAD^+^, a decrease in endogeneous O_2_^−^ causes reductive stress, and in turn activates Fur (ferric uptake regulator) [108]. Fur generally represses ion transport and ion siderophore biosynthetic genes when complexed with ferrous ion. Under ion limitation, ion dissociates from Fur, where Fur requires binding to Fe^2+^ to become active. O_2_^−^ deactivates Fur after its conversion to H_2_O_2_ by superoxide dismutase (SOD), through Fenton reaction (H_2_O_2_ + Fe^2+^ → HO + OH^−^ + Fe^3+^) [109]. Therefore, a decrease in endogeneous O_2_^−^ increases the availability of Fe^2+^, through a decrease in H_2_O_2_ level, and in effect activates Fur [110]. Namely, Fur senses the reductive stress and protect Fe-S clusters to be safe from damage by reactive oxygen species (ROS). It is essential for the cell to use iron economically, and this is attained by siderophores synthesis and iron transport regulation [111]. Iron transport and siderophores (e.g., enterobactin) pathway genes such as *talB* and *entF* are repressed by Fur [112,113,114], and enterobactin may be produced in *fur* mutant *E.coli* [115].

There are functional interactions between carbon and ion utilization *via* Crp and Fur, where many ion transport genes and several catabolic genes are subject to dual control. The *sodB* gene encoding SOD and *aceBAK* operon show opposite responses, being activated by the loss of Crp, and repressed by the loss of Fur, while such genes as *sdhCDAB, sucABCD*, and *fumA* genes in the TCA cycle are repressed by the loss of both TFs. Moreover, the loss of two TFs activates a heterogenous group of genes encoding Cfa, cyclopropane fatty acid synthase, and YggB, the mechanosensitive channel protein [116].

## 10. Growth-Phase Dependent Fis Expression

In *E.coli*, Fis (factor for inversion stimulation) is a nucleotide-associated protein, and is the most abundant during exponential growth phase [117]. Fis levels peak during early growth phase, and thereafter decrease until they become very low during stationary phase [118], where *fis* transcription is repressed by the stringent response [119], and *fis* is subject to growth rate control [120]. Moreover, the stringent control and the growth control all require the transcription factor DksA [120]. Fis seems to play a widespread role in signaling conditions of high nutritional control and outfitting the cells for efficient nutrient uptake, and rapid growth [118]. Fis also plays a role in signaling poor nutritional condition, where in response to amino acid starvation, *fis* is subject to severe and rapid negative control by the stringent response [120]. In relation to stringent response, (p)ppGpp and DksA interact with RNAP [121].

## 11. Stringent Response to Nutrient Starvation

The nutrient limitation may be defined as hunger and starved conditions, where the hunger state is defined to be in-between feast (nutrient excess) and famine (starvation or without nutrient) [122]. Bacteria generally have distinct strategies for the starvation in different nutrient sources. The individual hunger responses may be superimposed on a common protective starvation response [122].

Carbon limitation finally leads to amino acid limitation, which requires the signaling pathways *via* RelA and SpoT during carbon and amino acid limitation [123]. During stringent response, nutrient limitation leads to accumulation of ppGpp (guanosine 3',5'-bisphosphate) [124], which may bind to RNA polymerase [125], where ribosomal RNA and proteins are negatively regulated by ppGpp, which implies that protein biosynthesis declines, and in turn the cell growth rate decreases. During amino acid limitation, (p)ppGpp (including penta-phosphate pppGpp) is mediated by RelA. The accumulation of (p)ppGpp depends on the dual activity of SpoT as (p)ppGpp-hydrolase or ppGpp synthetase. SpoT is activated in response to fatty acid starvation, carbon source starvation, diauxic shifts, phosphate limitation, ion limitation, hyper-osmotic shock, and oxidative stress [126].

The alamone ppGpp is involved in the regulation of σ^S^ on the transcriptional and posttranscriptional level [127]. The elevation of σ^S^ negatively regulates σ^D^-dependent house keeping genes, such as TCA cycle genes [128]. Moreover, ppGpp influences the competition between different stress-related sigma factors in the binding of RNA polymerase core enzyme at the expense of σ^D^ [129] and RNA polymerase availability [123].

As implied before, Cra and cAMP-Crp coordinately regulate the main metabolic pathways, and these play important roles for a good economic balance between precursor formation for biosynthesis and energy generation during C-limitation [123]. Catabolite control involves also the regulation of chemotaxis. This indicates that cells pursue “offensive” strategy in order to exploit the low amount of available carbon sources [123]. On the other hand, ppGpp concentration increases with lower growth rates, and affects RpoS, and ppGpp accumulates immediately after onset of nutrient starvation. The nucleotide ppGpp regulates the ribosome concentration and the reduction of the cell growth rate. This might be regarded as “defensive” strategy, because ppGpp thereby regulates the withdrawal of precursors (and energy) from the central carbon metabolism [123].

## 12. Stationary Phase Regulation by RpoS

The culture condition changes from glucose-rich to acetate-rich conditions, and changes further to carbon-starved conditions in the batch cultivation. RpoS, the master regulator of the stationary phase or stress-induced genes regulates such genes as those for the carbohydrate PTS, *crr*, glycolytic pathway genes such as *fbaB* and *pfkB*, the acetate-forming gene *poxB*, the non-oxidative PP pathway genes such as *talA* and *tktB*, and TCA cycle genes such as *acnA* and *fumC*. In addition, some of the amino acid and fatty acid metabolic pathway genes such as a *rgH*, *aroM,* and *yhgY*, and energy metabolism genes such as *narY*, *appB,* and *ldcC* are also regulated in an *rpoS*-dependent manner [130,131,132,133,134,135]. As the cell utilizes glucose, acetate is produced as the major fermentative product under aerobic condition. Then, the cell utilizes acetate as a carbon source during early stationary phase of growth. When acetate is used up, the cell starts to utilize amino acids as carbon and nitrogen sources during the stationary phase. RpoS regulates the expression of many genes at the onset of stationary phase or carbon-starved conditions as well as other stress conditions in *E. coli* [127,133,134]. RpoS stimulates the expression of several oxidative stress response genes such as *katE*, *katG*, *sodC*, and *dps* and osmotic stress response genes such as *osmE*, and *osmY*. Strains lacking a functional *rpoS* gene also fail to express the genes for acid resistance such as *gadA* and *gadB*, near-UV resistance gene *nuv*, acid phosphatase genes *appAR* [127,131]. Under normal situation with roch medium, RpoS is rapidly degraded by ClpXP proteases, and the proteolytic activity of this enzyme is considerably reduced [127,133,134].

Although the roles of RpoS are originally described for various types of stress response, the regulatory roles of RpoS are not restricted to the stress response genes only. In *E. coli*, RpoS-dependent genes are found all over the chromosome, whose function ranges from DNA repair and protein synthesis to the transport, biosynthesis and metabolism of sugars, amino acids, and fatty acids. RpoS regulates the expression of DNA repair enzymes such as exonuclease encoded by *xth*A, methyl transferase encoded by *ada*, the gene that determines the cell morphology such as *bol*A, the genes encoding transport, and binding proteins such as *gabP* and *ugpEC* [132,136] 

## 13. Biofilm, Motility by Flagella, and Quorum Sensing

Biofilm formation is one of the important microbial survival strategies, where biofilm development involves attachment of bacteria to surfaces and cell-cell adhesion to form microcolonies. This is useful for the cell to protect against predetors and antibiotics [137]. The attachment of bacteria to abiotic and biotic surfaces is made by motility, proteinaceous adhesion, and a cell-bound polysaccharide such as PGA (poly-β-1,6-N-acetyl-d-glucosamine), where PGA is a cell-bound exopolysaccharide adhesion [137]. As mentioned before, Csr plays important roles for biofilm formation, where *pga* operon is involned in PGA formation and excretion, and it is negatively regulated by CsrA. As mentioned before, CsrA also negatively regulates c-di-GMP, a second messenger involved in biofilm formation and motility [138]. Curli are extracellular proteinaceous structures extending from the cell surface for attachment during biofilm development [139]. Curli filaments are activated by CsgD, where it is inversely correlated with flagella synthesis. The master regulator of flagella synthesis is FlhD_2_C_2_, which activates the genes involved in motility and chemotaxis [140]. McaS (multi-cellular adhesion sRNA) represses CsgD expression, while activates FlhD and PgaA [140], and thus regulates the synthesis of curli flagella and polysaccharide.

As mentioned in the previous section, RpoS plays an important role during the stationary phase. The *csrA* expression is also under control of RpoS [141]. Moreover, biofilm formation is under catabolite repression by cAMP and Crp [142].

Quorum sensing is a cell-to-cell communication [143], where the signal molecules are homoserine lactones (AHL) synthesized by LuxI-type enzyme. At high cell density cultivations, LuxR-type regulator plays a role for the positive feedback in association with AHL when its concentration exceeds a threshold level [8]. The quorum sensing is considered to be the sensing of cell density, where in *E.coli*, CyaR represses *luxS* gene which encodes autoinducer-2 synthase [11].

## 14. Death Phase and Long-Term Stationary Phase Metabolism

After the stationary phase in the batch culture, the death phase and long-term stationary phase follow [144]. During the stationary phase, nutrient becomes exhausted, and waste products gradually accumulate, which may become a stress to the cell, and this eventually leads to the death phase in which the number of viable and culturable cells declines. Since the majority of the cells in the death phase are viable but non-cultuable or die, nutrients from a portion of such cells are released into the medium. The released nutrients support the survival of the remaining culturable cells, and viable and culturable cells can survive for months or years in the long-term stationary phase [145,146]. The σ^E^-dependent cell lysis is to eliminate damaged cells in the stationary phase in *E.coli* [146], where the cell lysis proceeds in the cascade of σ^E^ → expression of *micA* and *rybB* → reduction in Omp proteins in the outer membrane → disintegration of outer membrane [147]. The cell lysis cascade appears to be related to oxidative stress in the early stationary phase [148].

## 15. Effect of Oxidative Stress on the Metabolism

The microbial cell responds to oxidative stress by inducing antioxidant proteins such as superoxide dismutase (SOD) and catalase, where those are regulated by OxyR and SoxR [149]. SoxR is a member of the MerR family of metal-binding transcription factors, and it exists in solution as a homodimer with each subunit containing a [2Fe-2S] cluster. These clusters are in the reduced state in inactivated SoxR, and their oxidation activates SoxR as a powerful transcription factor [150]. The active form of SoxR activates transcription of *sox* gene, and its product, SoxS, belongs to the AraC/XylS family of DNA-binding transcription factors. Oxygen derivatives such as superoxide (O_2_^−^), hydrogenperoxide (H_2_O_2_), and the hydroxyl radical (OH) are usually generated as toxic by-products of aerobic metabolism in a cascade of monovalent reductions from molecular oxygen. Although these are not so reactive *per se*, O^−^ and H_2_O_2_ cause severe cell damage. H_2_O_2_ along with Fe^2+^ *via* the Fenton reaction produces OH, which reacts with any macromolecule such as protein, membrane constituents, and DNA [151,152]. O_2_^−^ exacerbates the Fenton reaction by increasing the intracellular pool of free iron, for instance, by releasing iron from O_2_^−^-oxidized [4Fe-4S] clusters. Despite their toxicity, reactive oxygen species (ROS) at low concentration are involved in the cell’s life and, therefore, should not be entirely eliminated. Potent basic defense systems maintain ROS at harmless levels but cannot deal with sudden increases in ROS production as oxidative stress.

The two enzymes involved in the oxidative PP pathway, G6PDH and 6PGDH that produce NADPH for biosynthesis, are significantly affected in both *soxR* and *soxS* mutants [153]. The activities of G6PDH and 6PGDH decrease in *soxR* and *soxS* mutants, compared to the parent stain. The down regulations of these two enzymes agree with slower growth rates in both mutants, since these enzymes are under growth rate-dependent regulation [154]. The down regulation of *zwf* gene in both mutants is also due to the effects of *soxS* and *soxR* genes deletion, since *zwf* is a member of *soxRS* and multiple antibiotic resistance (*mar*) regulons. Unlike *gnd*, *zwf* expression is transcriptionally activated by SoxS for oxidative stress [152,155] (Supplementary Figure S1). The *pntA* (membrane bound transhydrogenase) transcripts which is involved in NADPH generation from NADH [156], are up-regulated in both *soxR* and *soxS* mutants to backup the reduced NADPH production in these mutants, since NADPH plays a significant role to reduce oxidative stress [156].

**Figure 6:**
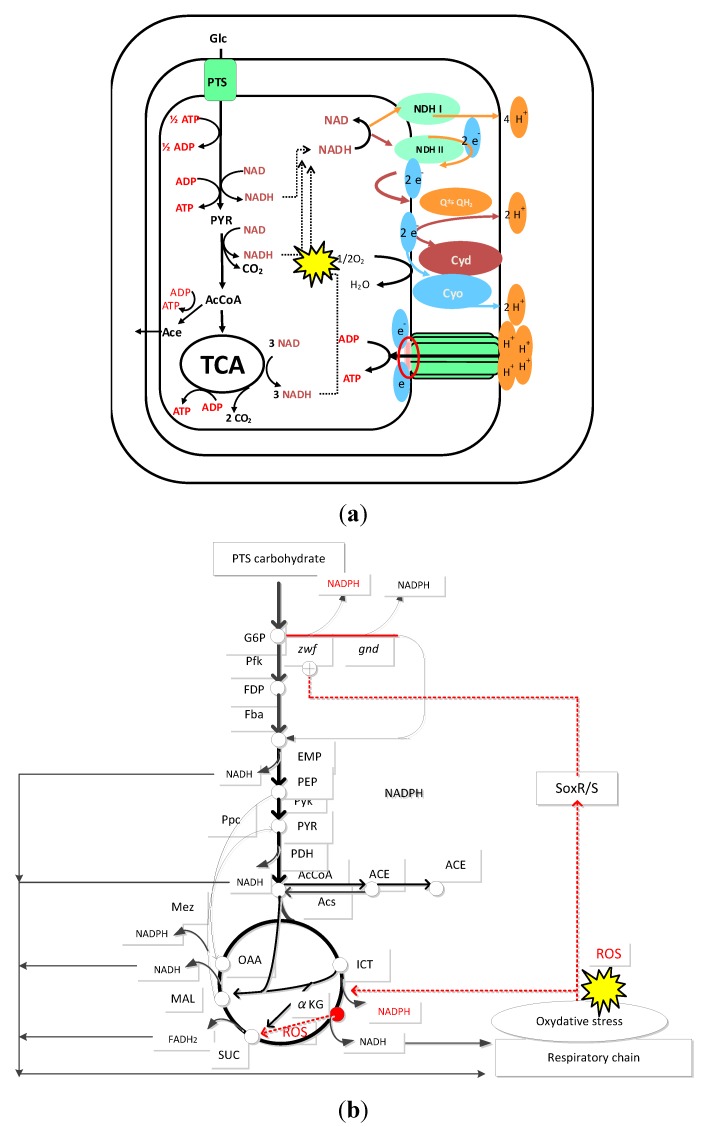
(**a**) Oxidative stress in the respiration; and (**b**)metabolic regulation for NADPH production.

As the specific glucose consumption rate increases, and the respiration increases, SoxR/S is activated as implied in Figure 4a. Then, SoxS activates *zwf* gene expression (Figure 4d), where the activation of the oxidative PP pathways may be due to NADPH production for oxidative stress as well as for the cell synthesis (Figure 6).

In general, all aerobic organisms produce NADH and FADH_2_ in the TCA cycle, where these reducing equivalents are oxidized in the respiratory chain, and the electrons generated from the reducing equivalents are subsequently transferred to cytochromes where O_2_ is converted to H_2_O. The ATP is then generated by the proton motive force (PMF) with ATPase (Figure 6a). Although this process is universal among all aerobic organisms, inefficient electron transfer *via* the respiratory complexes results in one electron reduction of diatomic oxygen, a phenomenon known to generate toxic ROS [157]. Since NADPH plays an important role for detoxification of ROS, some prokaryotic microorganisms such as *E.coli* produce NADPH at ICDH in the TCA cycle together with the reactions at G6PDH, 6PGDH, and possibly at Mez (Figure 6b). As the oxygen level decreases with reduced activity of the TCA cycle, less ROS is generated, and the NAD(P)H generated at ICDH varies in general [157].

αKG is a key participant in the detoxification of ROS such as H_2_O_2_ and O_2_^−^ with concomitant formation of succinate, where it is a biomarker for oxidative stress [157] (Figure 6b). Moreover, NADPH producing ICDH is activated, while NADH producing KGDH is deactivated in the cultivation of *Pseudomonas fluorescens* [157]. This indicates that for both prokaryotic and eukaryotic cells, the TCA cycle acts both as a scavenger and generator of ROS, and its modulation is important for regulating intracellular ROS (Figure 6b). Note that, unlike ROS detoxifying agents such as SOD and catalase, which only decompose ROS without affecting their production [158], the TCA cycle can regulate both their formation and decomposition. The concomitant accumulation of succinate may act as a potent signal for this [159] (Figure 6b).

## 16. Acid Shock Response

The cell such as *E.coli* has the regulation systems in response to acidic condition [159,160,161,162]. Some of these depend on the available extracellular amino acids such as glutamate, arginine, and lysine, where the intracellular proton is consumed by the reductive decarboxylation of the amino acid followed by the excretion of 𝛾-amino butyric acid (GABA) from cytoplasm to the periplasm by the anti-porter that also imports the original amino acid [159]. *E. coli* is acid resistant by such genes as *gadAB* which encodes glutamate decarboxylase and *gadC* which encodes glutamate/GABA anti-porter. Glutamate decarboxylase production increases in response to acid, osmotic, and stationary phase signals. The *gadA* and *gadB* genes for glutamate decarboxylase isozymes form a glutamate-dependent acid response system, where the process of decarboxylation consumes an intracellular proton and helps maintain pH homeostasis. There exists similar acid resistant systems for the case of using arginine instead of glutamate by arginine decarboxylase, where the antiporter is AdiC in this case [163,164], and for the case of using lysine by lysine decarboxylase [164]. The cells grown in media rich with amino acids such as LB are acid resistant [159].

In the typical batch culture, organic acids are mostly accumulated at the late growth phase or the stationary phase, and GadA and GadB proteins increase in response to the stationary phase and at low pH [165]. RpoS, which increases at the late growth phase and the stationary phase as well as Crp, is involved in acid resistance [159]. As implied by the involvement of Crp, the acid resistant system is repressed when glucose is present. Moreover, ATPase is involved in this system [162], where ATPase is mainly utilized for the protons in the periplasm move into the cytosol across the cell membrane producing ATP from ADP and P_i_ by the negative proton motive force (PMF). Since the basic problem of acid stress is the accumulated proton in the cytosol, this proton may be pumped out through ATPase by hydrolyzing ATP with reversed proton move due to positive PMF at low pH such as pH 2 or 3 [162]. Without amino acid in the media, this acid response system is activated by utilizing ATPase [161,166], where the positive PMF pumps extra protons (H^+^) from the cytoplasm with consumption of ATP [162]. Namely, PMF is operated in the reverse direction as compared to the case of producing ATP.

The acidic pH lowers cAMP levels in exponentially growing cells in the minimal glucose medium. Since cAMP-Crp represses RpoS, this may elevate RpoS, and increases the expression of *gadX*. However, GadW represses RpoS synthesis at acidic condition, and in turn GadX synthesis. GadX, when not repressed by GadW, is acid induced due to changes in cAMP. GadW is also acid-induced when it is not repressed by GadX. GadX directly binds to the *gadW* promoter region. The overall regulation system seems to be quite complex, involving EvgS/A, B1500, PhoQ/PhoP [167].

As explained before, the two-component system of EnvZ and OmpR regulates porin expression, where OmpR may be a key regulator for acid adaptation, and thus *ompR* mutant is sensitive to acid exposure [152]. The acid-inducible *asr* gene is regulated by the two-component system PhoR/B, and thus *phoR/phoB* deletion mutant fails to induce *asr* gene expression [168].

## 17. Heat Shock Response

The organisms respond to a sudden temperature up-shift by increasing the synthesis of a set of proteins. This phenomenon is called as heat shock response, where this does not restrict to the temperature up-shift, but also other stresses, as will be mentioned later in the section of solvent stress. The heat shock proteins play important roles in the assembly and disassembly of macromolecular complex such as GroE, the intracellular transport such as Hsp70, transcription such as σ^70^, proteolysis such as Lon, and translation such as lysyl tRNA synthetase. The heat shock response in *E. coli* is mediated by Eσ^32^, where E denotes RNA polymerase holoenzyme. Among them, *groEL, dnaK*, and *htpG* encode major chaperones such as Hsp 60, Hsp 70, and Hsp 90. ClpP, Lon, and HtrC are involved in the proteolysis. DnaK, DnaJ, GrpE, and RpoH are involved in the autoregulation of heat shock response. DnaK prevents the formation of inclusion bodies by reducing aggregation and promotion of proteolysis of misfolded proteins. A bichaperone system involving DnaK and ClpB mediates the solubilization or disaggregation of proteins. GroEL operates protein transit between soluble and insoluble protein fractions and participates positively in disaggregation and inclusion body formation. Small heat shock proteins such as IbpA and IbpB protect heat-denatured proteins from irreversible changes in association with inclusion bodies [169,170].

Hoffmann *et al.* [171] investigated the metabolic adaptation of *E. coli* during temperature induced recombinant protein production, and showed that cAMP/Crp- controlled LpdA of pyruvate dehydrogenase complex (PDHc) and SdhA in the TCA cycle are highly induced. In *E. coli*, heat shock protein synthesis rates peak at about 5–10 min after the temperature upshift and then decline to a new steady-state level [172]. σ^70^ is itself a heat shock protein, and the increase in its concentration after heat shock may contribute to its decline in heat shock protein synthesis. DnaK contributes to the shutoff of the high level synthesis of heat shock proteins [173].

Upon heat shock, *crp* gene expression increases, and *lpdA* gene expression follows the similar pattern. Moreover, *mlc* gene expression follows the similar pattern as that of *rpoH*. Eσ^32^ plays an important role in balancing the relative concentration of Mlc and EIICB in response to the availability of glucose in order to maintain inducibility of Mlc regulon at higher temperature [174]. The *mlc* gene is transcribed by two promoters, P_1_ and P_2_, and has a binding site of its own gene product, where P_2_ promoter could be recognized by RNA polymerase containing the heat shock sigma factor.

Let us consider the production mechanism of acetate at higher temperature. Acetate excretion occurs through Pta-Ack pathway, or may possibly by Pox pathway. Acetate utilization occurs through Acs. Transcription occurs from two σ^70^-dependent promoters such as distal promoter *acs* P_1_ and proximal promoter *acs* P_2_ [175,176]. Crp functions directly as the critical transcription factor. Cells control the acetate switch primarily by controlling the initiation of *acs* transcription from the major promoter *acs* P_2_ [175,177]. Activation of *acs* transcription depends on cAMP-Crp (Supplementary Figure S1). The cAMP-Crp binds two sites within the *acs* regulatory region. However, Fis and Ihf independently modulate Crp-dependent activation of *acs* P_2_ transcription [178]. As such, the activation of *crp* may cause *acs* to be upregulated, where *acs* gene is also under control of RpoS [179].

The respiration is activated during the temperature upshift [171], and superoxide dismutase gene (*sod*) is induced in response to the oxidative stress imposed by dioxygen or by the redox active compounds such as viologens or quinones caused by the temperature up-shift [180].

## 18. Tolerance to Various Stresses in Biofuels Production by Microorganisms

The biofuels production by microorganisms has been paid recent attention. However, many biofuels are toxic to microorganisms, and reduce the cell viability through damage to the cell membrane and interference with essential physiological processes. Several attempts have been made to improve the tolerance to biofuels, where biofuel export systems, heat shock proteins, and membrane modifications have been considered [181]. The effect of biofuels on the cell is through hydrophobicity of the cytoplasmic membrane, where the accumulation of solvent in the cytoplamic membrane increases permeability of membrane, diminishes energy transduction, interferes with membrane protein function, and increases fluidity [181,182,183,184]. This may cause the release of ATP, ions, and phospholipids, RNA and proteins, and thus the cell growth is depressed due to disturbances on ATP production by diminished proton motive force (PMF). Moreover, the increase in fluidity affects the nutrient transport as well as energy transduction.

Toxicity levels vary depending on the microbes and the types of biofuels and biochemicals. In general, longer chain alcohols are more toxic than short chain alcohols. Efflux pumps are membrane transporters that recognize and export toxic compounds from the cell by PMF, where this is important for the cell to survive by exporting bile salts, antimicrobial drugs, and solvents. The *acrAB-tolC* pump in *E.coli* provides tolerance to hexane, heptanes, octane, and nonane [185]. Efflux pumps are effective for increasing tolerance and production of biofuels, in particular, for long chain alcohols such as alkanes, alkenes, and cyclic hydrocarbons, but those are not effective for exporting short chain alcohols such as 1-propanol and isobutanol [186].

The heat shock proteins are up-regulated in response to short chain alcohols [187], where the sigma factor for heat shock such as RpoH is activated [110], and heat shock and protein refolding genes such as *rpoH, dnaJ, htpG,* and *ibpAB* are up-regulated [188], while *groESL, dnaKJ, hsp18, hsp90* are p-regulated in *Chrostridium acetobutylicum* [189]. Over-expression of heat shock proteins may increase tolerance against biofuels [190,191].

In general, solvents disrupt the cell membrane structure and have a strong impact on physiological function, and eventually leading to the cell death [192]. To overcome this problem, solvent tolerant microbes change the composition of the fatty acids from *cis* to *trans* unsaturated fatty acids catalyzed by *cis*-*trans* isomerase (*cti*), thus decreasing membrane fluidity, preventing the entry of solvents into the cell [193,194]. In addition, modifications to phospholipid headgroups or phospholipid chain length increase solvent tolerance [187].

In relation to solvent stresses caused by the accumulation of biofuels in the culture broth, the primary role to protect the cell from such stress is made by outer membrane porin proteins. Since cytosolic membrane is also under stress condition, respiration and membrane proteins as well as general stress response mechanism are affected [184]. Several transcription factors are affected by isobutanol in *E.coli* [110]. The reactive oxygen species (ROS) highly increase in response to the stress caused by n-butanol in *E.coli* [188]. Moreover, the improved tolerance against n-butanol can be made by over-expression of ion transport and metabolism genes such as *entC* and *feoA*, as well as acid resistance-related gene *astE* and inner membrane protein gene *ygiH* [191].

In order to keep pH constant, alkali such as NaOH is supplied during the cell growth in practice, which results in the increase in sodium ion (Na^+^), where *nhaA* gene encoding Na^+^/H^+^ antiporter membrane protein and *nhaR* gene encoding the NhaA regulatory protein can be overexpressed in *pflB* mutant, showing performance improvement for lactate fermentation [195].

RpoS plays important roles in response to the stresses caused by the accumulation of biofuels [110,196]. Some of the global regulators such as ArcA, Fur, and PhoB are activated, probably indirectly, by isobutanol [110].

## 19. Concluding Remarks

As seen above, the global regulators are responsive to the specific stimuli. Examples of such pleiotropic TFs in *E.coli* are Crp, a primary sensor for C-availability, NtrBC, a sensor for N-availability, PstSCAB and PhoR, the sensor for P-availability, CysB, the sensor for S-availability, and Fur, the sensor for ion availability. Functional interactions among such regulators must coordinate the activities of the metabolon so that the supply of one type of nutrient matches the supply of other nutrients [197]. Thus, multiple links between C and N metabolism has been identified [77,198]. Other functional links between C and S metabolism [199], and between C and ion metabolism [116,200] have been identified. Moreover, the links between S and N limitations are also identified [90].

In general, bacteria in nature live far away from the optimal growth conditions, where multiple stresses are imposed to the cell. Therefore, the cell must have the ability to sense, integrate, and respond to a variety of stresses for survival. Although little is known about “cross-stress” protection, cross stress dependencies are ubiquitous, highly interconnected, and may emerge within short time frames [201]. In *E.coli,* pre-adaptation to C or N limitation increases survival rates after heat shock or oxidative stress [202], where a possible link may be made by a heat shock regulator, RpoH [203]. Crp may play some role in this, since it is commonly involved in the above stresses. In fact, a high degree of overlap was observed in the transcriptional profiling for different stresses such as starvation, osmotic and acidic stresses [136], where high osmolarity and high temperature induces the oxidative stress regulons such as SoxRS and OxyR [204,205]. The responses to n-butanol share the same high overlap with those in heat shock, oxidative, and acidic stresses [188].

As mentioned in this article, the specific metabolites such as FDP, PEP, PYR, and αKG in the main metabolic pathways play important roles for metabolic regulation. This implies that these metabolites might play roles for the coordinated and integrated metabolic regulation. Note, however, that metabolomics alone is inadequate to understand cellular metabolic activity [206]. It is quite important to correctly understand the metabolic regulation in response to nutrient starvation and culture environmental stresses by integrating different levels of information such as fluxes, protein expression, gene expression as well as metabolite concentrations. What we understand the metabolic regulation mechanism so far is limited, and it may be important to get deep insight into the whole cellular metabolic systems, and apply this for the next generation metabolic engineering.

## Abbreviations

PP pathway: pentose phosphate pathway
TCA cycle: tricarboxylic acid cycle
PTS: phosphotransferase system
EI: enzyme I
EII: enzyme II
HPr: histidine-phosphorylatable protein
Cya: adenylate cyclase
CIT: citrate
E4P: erythrose-4-phosphate
FDP: fructose-1,6-bisphosphate
F6P: fructose-6-phosphate
G6P: glucose-6-phosphate
GAP: glycelaldehyde-3-phosphate
GLC: glucose
GOX: glyoxylate
ICI: isocitrate
KDPG: 2-keto-3-deoxy-6-phosphogluconate
αKG: α-ketoglutarate
MAL: malate
OAA: oxaloacetate
PEP: phosphoenol pyruvate
6PG: 6-phosphogluconate
PYR: pyruvate
R5P: ribose-5-phosphate
RU5P: ribulose-5-phosphate
S7P: sedoheptulose-7-phosphate
SUC: succinate
X5P: xylulose-5-phosphate
Ack: acetate kinase
Acs: acetyl coenzyme A synthetase
Adk: adenylate kinase
Ald: aldolase
CS: citrate synthase
Eno: enolase
Fum: fumarase
G6PDH: glucose-6-phosphate dehydrogenase
GAPDH: glyceraldehyde-3-phosphate dehydrogenase
ICDH: isocitrate dehydrogenase
Icl: isocitrate lyase
LDH: lactate dehydrogenase
MDH: malate dehydrogenase
Mez: malic enzyme
MS: malate synthase
Pck: phosphoenolpyruvate carboxykinase
PDH: pyruvate dehydrogenase
Pfk: phosphofructokinase
PGDH: 6-phosphogluconate dehydrogenase
Pgi: phosphoglucose isomerase
Pgk: phosphoglycerate kinase
Ppc: phosphoenolpyruvate carboxylase
Pps: phosphoenolpyruvate synthase
Pta: phosphotransacetylase
Pyk: pyruvate kinase
Ru5P: ribulose phosphate epimerase
R5PI: ribose phosphate isomerase
SDH: succinate dehydrogenase
SOD: superoxide dismutase
Tal: transaldolase
Tkt: transketolase

## Supplementary Files

Supplementary File 1 Supplementary File (PDF, 229 KB)
