# Supplementary material for: Regulation Systems of Bacteria such as Escherichia coli in Response to Nutrient Limitation and Environmental Stresses
Source: Metabolites. 2013 Dec 30;4(1):1–35. doi: 10.3390/metabo4010001 (PMC4018673; doi:10.3390/metabo4010001)
Supplement: Supplementary File 1 — Supplementary File (PDF, 229 KB) [file metabolites-04-00001-s001.pdf]

Supplementary File

Table S1. Effect of global regulators on the metabolic pathway gene expressions.

Global regulator	Metabolic pathway genes
Cra	+: *aceBAK*, *cydB*, *fbp*, *icdA*, *pckA*, *pgk*, *ppsA*<br>−: *acnB*, *adhE*, *eda*, *edd*, *pfkA*, *pykF*, *zwf*
Crp (cAMP-Crp)	+: *aceEF,acnAB,acs*, *focA*, *fumA*, *gltA*, *lpdA*, *malT*, *manXYZ*, *mdh*, *mlc*, *pckA*, *pdhR*, *pflB*, *pgk*, *ptsG*, *sdhCDAB*, *sucABCD*, *ugpABCEQ*,<br>−: *cyaA*, *lpdA*, *rpoS*
ArcA/B	+: *cydAB*, *focA*, *pflB*<br>−: *aceBAK*, *aceEF*, *acnAB*, *cyoABCDE*, *fumAC*, *gltA*, *icdA*, *lpdA*, *mdh*, *nuoABCDEFGHIJKLMN*, *pdhR*, *sdhCDAB*, *sodA*, *sucABCD*
Mlc	−: *crr*, *manXYZ*, *malT*, *ptsG*, *ptsHI*
PdhR	−: *aceEF*, *lpdA*
CsrA	+: *eno,pfkA,pgi,pykF,tpiA*<br>−: *fbp,glgC, glgA, glgB,pgm, ppsA,pckA*,
Fur	+:<br>−: *entABCDEF, talB,sodA*
RpoS	+: *acnA, acs, ada, appAR, appB, argH, aroM, dps, bolA, fbaB, fumC, gabP, gadA, gadB, katE, katG, ldcC, narY, nuv, pfkB, osmE, osmY, poxB, sodC,talA, tktB,ugpE,C, xthA, yhgY,*<br>−: *ompF*
SoxR/S	+: *acnA, cat, fumC, fur, sodA, sox, zwf*<br>−:
OxyR	+ : *ahpC, ahpF, katG*<br>−:
PhoR/B	+: *phoBR, phoA-psiF, asr, pstSCAB-phoU*<br>−: *phoH, phnCHN, ugpA, argP*
Fnr	+: *acs, focA*, *frdABCD*, *pflB*, *yfiD*<br>−: *acnA*, *cyoABCDE*, *cydAB*, *fumA*, *fnr*, *icdA*, *ndh*, *nuoABCEFGHIJKLMN*, *sdhCDAB*, *sucABC*
